# Epistemic responsibility in the face of a pandemic

**DOI:** 10.1093/jlb/lsaa033

**Published:** 2020-05-28

**Authors:** Neil Levy, Julian Savulescu

**Affiliations:** University of Oxford, Uehiro Centre for Practical Ethics, Oxford, UK

**Keywords:** Responsibility, knowledge, testimony, deference

## Abstract

Should non-experts defer to epidemiologists with regard to the response to the coronavirus pandemic? We argue that deference is required with regard to settled science: non-experts (that is, people who may possess expertise of their own but whose expertise is not relevant to a particular question) ought to defer with regard to climate science and the efficacy of vaccines. However, we suggest that this deference is warranted because these questions have been appropriately probed many times by many different kinds of people. While non-experts should defer to epidemiologists with regard to matters within the sphere of epidemiology specifically, responding to the pandemic requires expertise from many fields. We best build a consensus worth deferring to by contributing our expertise now. Ethicists and philosophers are not epistemically arrogant if they question policy responses. Rather, they play a responsible role in building a reliable consensus.

The COVID-19 pandemic currently wracking the world represents a crucial test for our ethical toolkit. Governments, institutions, and individuals are suddenly called upon to make life and death decisions for which they typically ill-prepared. Vocabulary that has suddenly become so familiar—‘flatten the curve’, ‘social distancing’, and ‘R0’—was unknown to most of us a bare few weeks ago. Even for experts, every option continues to have huge uncertainties associated with it. When experts are divided and unsure, how are the rest of us responsibly to decide how to act and who to trust?

In this paper, we focus on the responsibilities, in particular, of people like ourselves and (we assume) many readers of this journal: people with a genuine claim to expertise in some area, but none in epidemiology. They may be experts in ethics or in law, or in some other branch of the sciences or of medicine. These kinds of expertise are (we believe) every bit as valuable as expertise in epidemiology, but they usually leave us ill-equipped to assess claims in that discipline. What is our role and what are our responsibilities in the face of our epistemic limitations? How do our responsibilities differ from those of other nonepidemiologists (politicians and businesspeople, for example) whose decisions shape responses to the pandemic?

In this paper, we will argue that in the face of settled science, nonepidemiologists fulfill their epistemic responsibilities (mainly, at any rate) by deference. We should defer to the science, because we lack the capacity to second guess it and because it is reliably formed. But when science is not yet settled, epistemic responsibility does not require such deference. A scientific consensus is reliable because many different individuals with many different kinds of expertise have played a role in stress testing and in contributing to it. There is no reliable consensus on the coronavirus pandemic as yet, and epistemic responsibility requires putting it to the test, not deferring. Ethicists, philosophers, and other nonepidemiologists can play an important role in this stress testing, and how we shoulder this burden will help to define the final toll of the virus, measured in lives and in wellbeing.

## I. RESPONSIBLE DECISION-MAKING IN NORMAL TIMES

Responsible action has demanding epistemic conditions.^1^ While there is extensive debate about what, precisely, an agent must know in order to be responsible for her actions, there is little question that ignorance can excuse. An agent who poisons a friend by putting arsenic in his coffee might be guilty of murder (if she knew it was arsenic), of manslaughter (if she did not know but ought to have checked—say because she knew the arsenic container and the sugar looked similar and were stored in the same cupboard, or even because she was so thoughtless as to store sugar and arsenic in the same cupboard), or may be entirely excused because she neither knew nor should have known that the sugar had been replaced by arsenic (whoever replaced it instead bears the blame).

There is an epistemic condition on action because only if we understand the nature of our actions and the kinds of effects they are likely to have are we able to exercise control over our behavior. The epistemic condition entails epistemic duties. To illustrate: in one variant of the case above, the agent is blameworthy because she should have gathered more information prior to acting. She should have checked whether the substance she was adding was sugar. As cases like this illustrate, agents may be derivatively responsible for an action (or omission) because they are directly responsible for an epistemic action: for whether (or how well) they carried out a prior obligation to gather evidence, to check sources, and to weigh reasons. It is important to note that epistemic actions are actions (consulting books or articles, googling, and asking other people), and the epistemic condition on action applies just as much as to these actions as any other. I am blameworthy for failing to check my sources, for example, only if I knew, suspected or should have known that I ought to doso.

The more significant an action, the greater our epistemic obligations (other things equal, of course). Few people do much research prior to purchasing a new dish cloth, but it would be irresponsible not to spend time learning about the area and carefully checking the house prior to taking out a large mortgage. Individuals therefore have significant epistemic responsibilities when it comes to their health and wellbeing and (perhaps even more) the health and wellbeing of those who are dependent on them. We expect parents to vet the people in whose care they leave their children, for example. Analogously, those who make decisions that can be expected to significantly impact on the health and wellbeing of others have heavy epistemic responsibilities to ensure that these decisions are appropriately informed. The greater our sphere of influence, determined by the number of people who are affected by our decisions, the degree to which they are affected, and their vulnerability, the weightier our epistemic responsibilities.

It follows from these principles that decision-makers have especially weighty epistemic responsibilities as we confront the COVID-19 pandemic. On the one hand, their decisions will affect the number of people who die from the virus (as well as, to some degree, the identity of those who die). On the other hand, the measures they put in place to limit deaths are costly, economically and socially, and these costs entail significant impacts on the health and wellbeing of those who survive the pandemic. For instance, the recession that seems certain to result from the shutdown of much of the economy across large parts of the world will itself be deadly. The recession that followed the 2008 financial crash is estimated to have resulted in at least 10,000 extra suicides in Europe and North America^2^ and more than a quarter of a million extra cancer-related deaths in OECD countries;^3^ at the time of writing, around 380,000 deaths globally have been linked to the virus. Feelings of isolation linked to the lockdown imposed in many countries will also take a toll on mental health.^4^ The economic impact and therefore (in all probability) the impact on mortality and morbidity of the recession induced by the shutdown are likely to be much greater than the 2008 recession.

While political leaders have the heaviest epistemic responsibilities, each of us has our own sphere of influence. Most of us have dependents or loved ones, whose health we affect by our decisions (for example, if we become infected their risk is likely to rise significantly). Interestingly, in many countries, individuals are explicitly exhorted to avoid infection not for their own sake but for the sake of others. There are two reasons medical authorities have emphasized our responsibility to others, rather than to ourselves. First, the most mobile and active members of the population appear to be at a relatively low risk of significant effects from the virus, with many having mild or no symptoms, but the coronavirus is highly infectious in the absence of measures to limit its spread. We therefore have a responsibility to avoid infection so that we do not transmit the virus, directly or via a chain of transmission, to vulnerable others. Second, authorities have emphasized the need to ‘flatten the curve’, that is, to ensure that the number of cases at any one time, and therefore the number of people requiring hospitalization, remains manageable. The lower the number at any one time, the smaller any shortfall in essential resources, like ventilators and trained personnel.

Since we are all decision-makers, we all face epistemic responsibilities, experts and nonexperts alike. But different individuals face different choices and have different epistemic responsibilities. The pandemic is not only the most urgent problem confronting us right now; it also has features that make it unique and entail that those nonepidemiologists who have expertise in other fields have very different kinds of epistemic responsibilities with regard to it than to other scientific (and medical) questions: conduct that would be irresponsible with regard to vaccinations, for example, may be obligatory with regard to the coronavirus.

## II. EPISTEMIC DEFERENCE

On many scientific questions, nonepidemiologists—including those with genuine expertise in some other area—fulfill their epistemic obligations (almost exclusively) by deference. One agent defers to another when the first accepts a proposition largely on the say so of the second. The most familiar kind of epistemic deference involves deference to testimony: explicit assertion with the aim of providing information. As philosophers have come to recognize, the use of testimony is absolutely necessary for successful navigation of the world.^5^ Much of what we know we know due to testimony. But epistemic deference extends much more broadly than explicit testimony.

Above, we cited the example of the parent who leaves her child in the care of someone else. We noted that the decision to do so entailed an epistemic obligation: to vet the carer. Were we living in Hobbes’ state of nature, this would be an extremely difficult obligation to fulfill. How would we decide whether another individual is trustworthy? In well-functioning contemporary societies, the obligation is relatively easy to fulfill. We leave our children with childminders who are in some way certified. Typically, government agents take over much of the burden for us. Inspectors ensure that the premises used are clean, that the staff have appropriate training and do not have criminal records (of a sort that would preclude them from such activities), and so on. So long as the parent is justified in having sufficient trust in these institutions, her obligation consists at most in ensuring that the child minder has the appropriate certifications (in fact, it may be even less demanding than that: she may be able to rely on the fact that any service that advertises openly will have been vetted and take their credentials on trust, at least in the absence of any red flags).

In relying on institutions to certify competence, we defer to them. We do this routinely, outsourcing much of our epistemic responsibility to others who are better placed than we are. We may not be able to assess someone’s teaching ability or commitment to children for ourselves, not without a great deal of effort and time, but others can take on the task for us. This kind of epistemic outsourcing is routine and is seen at all levels of decision-making. Political leaders are not in a good position to know the full range of facts relevant to their sphere of influence. For instance, they cannot be expected to have a good grasp of monetary policy and geopolitics and internet security and health economics, and so on, across a range of specialty subjects in which expertise is hard won. They, too, outsource their epistemic responsibilities. They rely on bureaucrats with appropriate training to produce economic forecasts, for example, and both bureaucrats and politicians seek expert advice from academia and industry.^6^

This kind of epistemic deference is required to compensate for our limitations as agents but is actually central to knowledge generation. Science works by distributing epistemic labor across groups of agents (within a lab, across labs and across whole fields). Science, as the stunningly successful enterprise it is, may be said only to truly begin when institutions of distributed cognition (like peer review) develop. Epistemic deference should not be as merely a corrective for our limitations but central to our success as epistemic agents.

However, our reliance on the testimony of others makes the problem of epistemic responsibility especially pointed.^7^ The greater our reliance, the less easily we can check for ourselves. In science, it is routine for people working in the same lab and on the same projects to be unable to verify each other’s work: they may lack the expertise to do so. For laypeople, this goes doubly: most of us are unable to verify for ourselves ‘that smoking causes cancer’ or ‘that the climate is warming’. Even those who have access to the scientific papers and sufficient scientific literacy cannot verify these claims for themselves: they can do so only by taking on trust that the background material cited is reliable, that opposing voices are not being filtered out for irrelevant reasons, and so on. Our need to trust sits uneasily with our obligation to believe responsibly.

We negotiate this dilemma by the use of markers of expertise. Just as the parent relies on certification by regulators to ensure that a potential childminder is competent and reliable, so policy makers utilize certification by institutions and other such markers. There is some debate over which markers of expertise we ought to use,^8^ but widespread agreement about the basic picture. We do, and should, utilize credentials (like degrees from reputable universities, membership of learned societies), track record (eg publications in high profile journals), intellectual honesty and independence (a reliable expert will declare industry funding and may be entirely independent of industry) (Table 1).

**Table 1:** Markers of expertise. We rightly give more weight to testimony when it comes from an individual who possesses such markers, so long as the testimony concerns their field of expertise

Credentials	Possession of a higher degree; institutional affiliation; membership of learned societies
Track record	Success at predicting events; publications in peer reviewed journals; awards and prizes
Intellectual honesty	Declarations of conflicts and funding sources; admissions of past errors
Argumentative capacity	Ability to counter arguments from rivals and to explain apparent anomalies
Consensus	Testimony from multiple experts outweighs testimony from a minority

These markers of expertise attach to individuals except the last: consensus. It is rational to prefer consensual testimony, other things being equal, because individuals are always subject to biases, but the perspective of multiple individuals may neutralize bias by ensuring that they are countered.^9^ Policy makers and individuals therefore should and do place particular weight on claims made not just by experts, but on statements that represent a consensus (or at any rate a majority opinion) among experts. For this reason, we rightly place great weight on the official positions of bodies that represent experts, like the National Academy of Science or the British Medical Association (interestingly, psychological work on children indicates that they utilize the same sorts of cues to decide between conflicting sources of testimony, preferring testifiers with good track records to those with bad or no track record, and ignoring testimony that conflicts with the consensus^10^).

## III. THE CORONAVIRUS: A PERFECT EPISTEMIC STORM

On many questions, policy makers and individuals have access to reliable information which they ought to use to guide their decisions. There is, for example, a consensus on the major environmental and lifestyle causes of cancer and on the efficacy and safety of vaccines. Responsible governments and individuals ought to be guided by this information and are often blameworthy if they ignore it or downplay its importance.^11^ Accordingly, we may blame parents who refuse to have their children vaccinated, for example.

Of course, many difficult questions remain unsettled: there is no consensus on the appropriate rate of taxation, for instance. Personal income tax rates vary widely across successful countries—from 57.2% in Sweden to 22% in Singapore, for example. There is a heated dispute among economists about the effect of higher taxation on incentives, and the extent of a reduction of incentives on overall tax take and on productivity. Those who make decisions about economic policy therefore cannot rely on a consensus for responsible decision-making. While our values (appropriately) play a role in all decision-making, in cases like this the degree to which values shape decisions may permissibly be much greater. Policies like this one are disputed, and these disputes are central to contemporary politics because at issue in such disputes are what kind of society we wish to live in. Even in cases like this, though, an expert consensus constrains the range of permissible options. Few economists would dispute that some possible tax rates on some income ranges are too high, and few would dispute that others are toolow.

Cases in which there is no consensus are typically and unsurprisingly cases in which feedback from implementing different options is relatively unreliable. Changing the rate of personal income taxation is changing one element in an incredibly complex, and open, system. It is therefore intrinsically difficult to distinguish signal from noise: without the capacity to control for other variables, observed effects may have multiple plausible explanations. Any genuine effect is likely to be small, relative to the combination of other factors in play. For this kind of reason, it is often the case that when decision-makers must make choices without guidance by an expert consensus, the stakes may not be as high as is often thought. The dispute between experts persists because different policy settings make a difference to outcomes that is difficult to detect. The stakes may also be lower than is often thought for another reason: policy decisions made by one government may be reversed by another at a later date (of course, we recognize that sometimes governments and individuals must make decisions which will have very large effects when an expert consensus is lacking; consider, for example, the decision by a parent whether to raise her child within a religion ornot).

In areas in which there is an expert consensus and expertise is hard to come by, responsible decision-makers and ordinary people defer. Anti-vaxxers are epistemically (and behaviorally) irresponsible because they do not have the expertise to contest the expert consensus. In the absence of such expertise, responsible agents are guided by the consensus. Similarly, climate change denialists are (almost always) epistemically irresponsible, because very few of them have the expertise to contest the consensus (of course, genuine experts who dissent can and do exist. They may not be epistemically irresponsible, but when the consensus is extremely strong—as it is in the case of climate science^12^—responsible nonexperts prefer the majority testimony).

The coronavirus crisis is different because there is not yet an expert consensus to which nonexperts can defer. Nor, however, is it a case in which the stakes are low, because decisions affect a variable that explains only a small part of the variance. For those decision-makers who must settle policy, it is a perfect epistemic storm. It presents these decision-makers with a challenge to which response is urgent, where an expert consensus is lacking but the different options can be expected to produce very large effects: to make a large difference to the number (and identities) of people who die. There is also no happy ending: large numbers of people will die and suffer no matter which option is adopted, though their numbers, identity, and timing of their deaths will differ.

There is, of course, no lack of expertise on infectious disease and on transmission. The science of epidemiology is well developed, and governments rightly have called upon its expertise. But the pandemic is caused by a novel coronavirus, and the mathematical modelling therefore must rely on assumptions that are at best reasonable. At the time of writing, we do not know what proportion of those infected will be asymptomatic (though a recent study of the general public in Iceland showed 13% carried the virus and 50% these were asymptomatic^13^), because testing for the virus has (understandably) been concentrated on those with symptoms and especially those in need of care. Because we do not know what proportion of those infected are asymptomatic, we do not know the infection fatality rate (that is, the proportion of those who contract the disease who will die from it) for the disease: basing our estimate on the numbers diagnosed alone will inflate the figure to an unknown degree.

Most countries have adopted social distancing and lockdowns in response to the pandemic. But assessing the relative costs and benefits of this intervention (relative to doing nothing and to less disruptive measures, such as selective isolation of the most vulnerable, making masks mandatory and advising social distancing) is very difficult, in the absence of anything like a good grasp on how harmful COVID-19 would prove were it left unchecked. We do not know what proportion of the infected would develop symptoms, nor how serious they would be. The vast majority of those who die with COVID-19 have at least one comorbidity (around half have three or more), making it difficult to attribute the cause of death.^14^ This same fact entails that some of those who die with the disease would have died around the same time and that the loss to them (measured in QALYs or even in life years without adjustment) is therefore relatively small. Accordingly, the toll of COVID-19 is better measured in excess mortality, rather than in raw numbers, and the excess mortality figures seem to paint a picture that is very different across different countries.^15^ For instance, while excess mortality has risen markedly in the UK (especially England), it is flat in Germany despite its 7500 deaths with the virus. Even the efficacy of physical interventions like lockdowns is contestable. We understand how viruses are transmitted and that understanding suggests that these kinds of interventions will be effective. But the gold standard review of the efficacy of social distancing and quarantine to slow the spread of respiratory viruses found small or uncertain effects.^16^

Unsurprisingly, given these uncertainties, surveys of epidemiologists reveal dramatic differences in their predictions of the future toll of the virus.^17^ For example, Sweden’s response is based on models that make very different predictions to those guiding the UK.^18^ Worse, we do not know what to expect when lockdowns end, as they inevitably must. Eradication of the virus seems unlikely to be achieved in any country other than New Zealand and perhaps Australia. Given that fact, when restrictions are eased the virus may take hold again.^19^ Lockdowns are often portrayed as an alternative to herd immunity, but until a vaccine is found they might better be seen as way of achieving herd immunity slowly, while keeping the pressure on the health system manageable (‘flattening the curve’). However, herd immunity is achievable only if infection confers (significant) future immunity—and the duration and extent of such immunity are currently unknown. Hence, lockdowns avoid a harm of a currently unknown magnitude, at a cost (measured centrally in terms of the direct and indirect effects of a severe recession on health and well-being) that is itself currently imponderable. On this basis, it has been suggested that the current response is more, perhaps much more, costly than can be justified.^20^

The debate between the mainstream, who advocate far-reaching interventions now, and those who urge that we wait until we have more information through conducting research, is a debate in important part about our epistemic responsibilities. Both sides accept that further information is very valuable, but one side thinks we already know enough to know that heavy handed intervention is warranted, either because we already know enough about the costs or because we know enough to know that the potential costs of false negatives (ie failing to implement such measures) are likely to be significantly greater than the potential costs of false positives.

In the face of these uncertainties, decision-makers might hope to be guided by the precautionary principle. Roughly, the principle advises that when we face a choice between options, we should always avoid any option that might result in grave harm.^21^ As we saw, the approach taken by those who urge lockdowns now might be justified on this basis. They might be represented as arguing that while the costs of lockdowns are known to be significant, in their absence we would likely see exponential growth in cases and a very large number of deaths (the Imperial College team whose modelling has centrally informed UK policy estimates that without interventions, COVID-19 would result in 40 million deaths globally^22^). As we have seen, however, these estimates build in questionable assumptions about infection fatality rates (IFRs) and about the efficacy of the interventions they urge. Apply the precautionary principle to models with different (plausible) assumptions—assume that the IFR is much lower than they think and physical barriers less effective than they suppose—and we get the opposite result: that we should act to avoid the truly grave harm of a devastating recession or depression.

While there are many different approaches nonepidemiologists—decision-makers and experts in other fields alike—might take, the following three seem to be the most widely advocated:

(i) Attempt to adjudicate the debate between the experts.
(ii) Attempt to split the difference between experts.
(iii) Attempt to identify which of the competing experts is more likely to be reliable.

None of these options is very palatable, but some are more irresponsible than others.

### A. Adjudicate the debate

We think this is the least palatable response to conflicting expert testimony. Laypeople, including those with genuine expertise in other arenas, are not well placed to assess the issues on which the conflict turns. They will usually be capable only of a big picture grasp of the relevant science and will almost always lack the highly specific and high level expertise that allows for not just an understanding of epidemiology, but an opinion that deserves respect on the dispute which divides the experts. Ordinary people are often subject to an ‘illusion of explanatory depth’:^23^ we take ourselves to have a much deeper grasp of the mechanisms at work in ordinary and scientific processes than we actually have (ironically, this illusion may itself arise from our facility in deferring to expert knowledge^24^). Because we suffer from this illusion, we are prone to think our capacity to assess complex issues for ourselves is much greater than it is. One of us has suggested elsewhere that the kind of epistemic arrogance that the illusion of explanatory depth sometimes gives rise to may cause laypeople to reject the expert consensus without justification.^25^

### B. Splitting the difference between rival experts

It is tempting to think that the responsible option for someone who is confronted with conflicting options that she knows to be recommended by genuine experts, but who is unable justifiably to decide between them, should split the difference. That is, she should adopt a policy that is midway between the two options. Something like this view might be supported by the conciliatory view in epistemology. Conciliationists maintain that when epistemic peers (people who have the same, or equivalently reliable, evidence and are equally good at assessing their evidence) disagree, both disputants should lower their confidence in their view.^26^ In the absence of another explanation for the dispute, each should think that at least one of the disputants has made a mistake, but that there is no more reason to think it is the other agent than themselves, and moderate their confidence accordingly. While conciliationists have not extended discussion to third parties, the natural upshot of their view is that such third parties, too, should hold a belief that is midway between the initial opinions.

However, while it is natural to think that third parties’ confidence should match that of the peers, it is a mistake to think that the confidence with which a belief should be held settles the content of the belief. Sometimes it might: as Galton showed, averaging the guesses of many different (equally expert, or nonexpert) individuals often produces a startlingly accurate estimate.^27^ However, on many questions there is no way to split the difference. The question might be a yes/no one (is this person guilty or innocent?), and sometimes splitting the difference would yield a response that is worse than either initial option, no matter which expert is right. Consider two physicians who disagree on whether a patient should be treated with antibiotics. They might both agree that giving her half the recommended dose of the antibiotics would be worse than giving her the full dose or nothing atall.

While it may usually or even always be appropriate to conciliate in the face of expert disagreement by lowering our confidence in our beliefs, it is often inappropriate to attempt to split the difference between the options recommended by competing experts. It may take expert knowledge to distinguish cases in which we ought to split the difference in this kind of way from those in which we should not (a layperson might indeed conclude that giving the patient half the recommended dose is the responsible way to proceed). Fortunately, the experts will sometimes be able to come to a consensus on whether such splitting would be worse than the rival views, even when they diverge considerably on which response would be best, and in such cases policy makers and individuals can defer to this expert consensus.

Is the current pandemic a situation in which such splitting might be warranted? Those epidemiologists who urge lockdowns argue that its effects are dose dependent. Nevertheless, there may be a threshold beyond which gains are marginal. No society has, or could, shut down its entire economy. Food production and distribution obviously must continue, for example. We might be able to shut down some sectors and not others and garner most of the benefits of a full lockdown. Whether a partial lockdown would produce a better balance of benefits and costs depends, in important part, on two factors. First, whether a partial lockdown would bring the R0 (the expected number of further infections directly generated by an infected person) below 1, and on the efficacy of so-called ‘track and trace’ as a means of controlling spread. If the R0 is above 1, the disease spreads exponentially and it will be hard to avoid overwhelming the health system. Only if track and trace (combined with self-isolation for those who have been exposed) is very effective could a nation hope to cope with an R0 above 1. Recent news from countries that have eased lockdowns suggests that these conditions are not satisfied. The partial easing in Germany appears to have caused the R0 to rise above 1 there,^28^ while cases in South Korea, which has the most aggressive track and trace system, seem to be rising rapidly too, prompting a return to social distancing.^29^ Preliminary evidence seems to tell against splitting the difference in this case, at least with regard to policy.

### C. Attempt to identify which of the competing experts is more likely to be reliable

This is the strategy that most writers on expertise seem to have recommended. One of us has expressed doubts about this strategy elsewhere,^30^ but whatever its merits in the kinds of cases typically at issue in these debates, their reflections are not helpful here. As we saw, philosophers like Alvin Goldman and Elizabeth Anderson have attempted to provide criteria for identifying trustworthy experts: that is, for distinguishing the reliable from the unreliable.^31^ In some ‘debates’, these criteria are called for: we need to distinguish the reputable climate scientist from those who are working in the service of the fossil fuel industry; the infectious disease expert from the crank who promotes misinformation. Misinformation about COVID-19 is proliferating, so these kinds of tests may be required. But distinguishing the crank from the genuine expert is not the dilemma that confronts us here. We are faced with deciding how to act in the face of conflicting testimony from genuine experts. Both sides are extremely well credentialed.

For instance, Neil Ferguson, whose modelling has been very influential on the UK government’s response to the virus, heads the Medical Research Council Centre for Global Infectious Disease Analysis at Imperial College London, while John Ioannidis, who is the best-known expert urging a less heavy handed response, is one of the most highly cited medical experts in the world (with an eye-watering H-index of 197). Criteria like prizes, independence and integrity, and track records do not seem useful in adjudicating disputes likethis.

We therefore conclude, tentatively, that the three strategies that might guide policy-makers and other nonepidemiologists are of little help here. In the absence of other strategies, we must make our decisions and calibrate our credences in other ways. How should we proceed? We focus first on policy-makers before turning to experts in other domains.

We think that expert consensus is the most important criterion that policy makers and individuals can use for responsible decision-making and we are confident that a consensus will emerge. But that fact, too, is of little help to those who must make decisions now. In fact, the consensus will probably emerge in large part as a consequence of these decisions: it will be assessing their impact and compared them to the impact of other policies (for instance, the less restrictive policies of Sweden) that experts will come to know with high confidence which is appropriate. Decision makers cannot wait for this information; rather, the information waits forthem.

In the absence of a reliable scientific consensus, government decision-making may be driven by other factors. One motivation for the actual, highly restrictive, response might be what we call the goalkeeper’s fallacy. There is some evidence that penalty kicks aimed down the middle of the goal are less likely to be saved than those aimed to the left or to the right.^32^ Part of the reason is that goalkeepers usually dive to the left or right to attempt to save the penalty. They do not stay upright and have a higher chance of saving the penalty because they believe (possibly rightly) that they will be blamed less if they made a spectacular and demanding, if futile, attempt to save the penalty than if they engage in the less demanding strategy of guarding the center. They have an incentive to dive, even if diving is less successful, on average, than not doing so. Similarly, governments may have an incentive to engage in spectacular interventions in the face of a public health crisis. The penalty, in terms of public opprobrium, for underreacting might be very much greater than the penalty for overreacting.

The pressure to be seen to be acting immediately and strongly is increased by a number of psychological biases to which both politicians and those who judge their performance are subject (Table 2). We are loss averse,^33^ putting greater weight on losses than forgone aims. Since counterfactual losses—the losses that we would have suffered had we chosen a different path—are not salient to us, we may tend to blame governments unfairly. But we are also temporal discounters,^34^ valuing the near term over the future. For that reason, it is politically and psychologically attractive to defer large losses. Economic and related health losses will occur in the further future while COVID deaths occur now. Finally, we suffer from salience biases and the availability heuristic:^35^ facts that are before us or brought easily to mind have a greater weight in decision-making than those that are pallid or seem theoretical. It is difficult to imagine cancer deaths related to 2008 crisis, but it is easier to see a person on a ventilator now.

**Table 2:** Psychological biases that may interfere with good decision-making in the context of the coronavirus. This is a very selective list; many others may play a role. Mitigating biases is difficult, but cost/benefit calculations, insofar as they are algorithmic, may help to reduce them (they are not a panacea because the inputs into such analyses may themselves be affected by bias)

Bias	Effect
Salience bias	Giving immediate harms undue weight
Affect heuristic	Preference for actions that are emotionally satisfying; might lead us to prefer saving lives of the ill over preventing abstract harms
Temporal discounting	Preference for saving lives/reducing harms now, versus those in the future
Confirmation bias	Tendency to look for evidence supporting our views (eg that it will save lives) and overlook evidence against it (that it might cause greater harms)
Illusory truth effect	Tendency to think that something that is fluently processed because it has been repeated multiple times (for instance) is more likely to be true
Mere exposure effect	Disposition to prefer the familiar to the unfamiliar
Omission bias	Disposition to see harms brought about by omission (eg failing to open businesses) as less serious than those brought about by actions

These kinds of factors make the position in which governments find themselves difficult. Faced with the absence of a consensus, the high stakes and immense pressure for immediate action stemming from both political and psychological considerations, we are reluctant to blame them for the restrictions that many have imposed (moreover, these restrictions may indeed turn out to be appropriate). They are under great pressure to act, arising from public pressure and political considerations. Moreover, they confront a genuine crisis: they face pressures that arise from the genuine and substantial risk of a human tragedy.

As we said above, in the face of settled science we believe that the epistemically responsible course of action is to defer to the consensus. While we have insisted that there are substantial uncertainties concerning the relative costs and benefits of different responses to the pandemic, it might nevertheless be thought that the responsible nonepidemiologist does best by taking a poll of epidemiologists and deferring to the majority. Perhaps a consensus is beginning to emerge already.

There are, however, reasons to distrust any emerging consensus. A reliable consensus emerges from what we called above the institutions of distributed cognition characteristic of science: peer review, the testing of hypotheses by multiple research groups, using different methodologies and with different biases, the replication of findings and the bringing to bear of expertise from a variety of different domains. All this takes time. While science has responded at dizzying speed to the pandemic, it is unlikely that these distributed mechanisms have had sufficient time for any consensus to emerge through virtuous mechanisms (as opposed to groupthink or self-silencing). As Eric Schliesser and Eric Winsberg have argued, ‘there is currently no well-ordered scientific community studying COVID-19 and its impact, so the emerging consensus could be the result of any number of all-too-human biases.’^36^ These differences between climate science and the state of knowledge concerning COVID-19 make an epistemic difference: there is no properly generated consensus to defer to in the lattercase.

While lockdown sceptics like John Ioannidis have urged that we gather more data before implementing potentially costly policies, deciding to wait before acting is itself potentially extremely costly. Governments cannot avoid making an extremely weighty decision. They cannot decide by adjudicating the issue or by deferring to (an appropriately generated) consensus. There may be some scope for splitting the difference, though that strategy, too looks unpalatable right now. We do not believe that there is any good response here. However, the least bad may be deference to particular experts.

Above, we noted that governments rely on advice from selected experts. To this end, most governments have formal or semi-formal procedures for generating advice: they have councils of expert advisors, or they commission reports from scientific academies, and so on. We believe that these policies are themselves epistemically responsible, when the selection procedure is well-designed (and not unduly subject to narrowly partisan bias). In the absence of any better method of guiding their decision-making, governments fulfill their epistemic responsibilities when it is guided by these established bodies or via these established channels. When there has not yet been time for a reliable consensus to emerge, dependence on these sources is risky: they may reflect only one or some sides of a multisided debate. But given that all strategies are very risky, and that the policy of deferring to experts is itself one that is valuable, we think such an approach is advisable. We note that following this policy might lead different governments to different approaches. We do not take this fact to be a mark againstit.

We turn now to the epistemic responsibilities of people like ourselves: experts in other domains but without the expertise to contribute directly to debates within epidemiology. Our responsibility is much less heavy: we do not bear the burden of making decisions that will have large effects on the numbers of people who die. Nevertheless, we have significant responsibilities, we suggest. In fact, we have epistemic obligations. While we ought to defer to settled science, when science is not settled, we have an obligation to questionit.

Again, let us contrast the science of COVID-19 to climate science. Many issues within climate science are entirely beyond the purview of the philosopher or the lawyer. But climate science, insofar as it is policy-relevant, extends far beyond the relationship between CO_2_ and temperature. It encompasses effects on human health, on well-being, on regulatory frameworks, and so on. It encompasses, that is, the nature of appropriate responses to the climate crisis. While it would be false to say that there is a consensus on these questions that is anything like as broad and deep as the consensus on the core scientific issues, nevertheless there is something approaching a consensus on the broad outlines of a response: on the need for a dramatic reduction in the production of CO_2._

This consensus has emerged only because lawyers and economists and even philosophers^37^ have played a role. That is, climate science presents us with a consensus which has already been tested and retested multiple times, for many decades. A great variety of experts from a great variety of disciplines have already had their say. The coronavirus pandemic, too, requires input from beyond the sciences. Identifying the appropriate response or responses to the pandemic is not a matter for epidemiologists alone: rather, it is a policy question, on which multiple different kinds of expertise bears. Epidemiologists are not experts in economics or in mental health or social policy or politics or behavioral science, and all these disciplines are relevant to the right response. If we are to have a reliable consensus view on the pandemic, we cannot defer to the science; not now. Just the opposite: the science will be reliable when it has been appropriately stress-tested, and that requires input from multiple perspectives right now. There is a role right now for ethicists, among many others, to put the science to the test. It’s not because the coronavirus is different from climate science that it is appropriate for people to second guess the science. It’s because it’s the same: there was a time when such second guessing was appropriate for climate science too. With regard to climate science, that time has long passed, not so with regard to the pandemic.

Ethicists are not epidemiologists and should not presume to cast doubt on matters that are within the specialist purview of that discipline (modelling, however, is not the purview of epidemiology: many different disciplines are competent to question mathematical models). But they have expertise that is relevant. Epidemiological models are not value-free: they embody assumptions about the value of lives and these assumptions are contestable. For instance, the models on which the UK relies count the value of all lives equally. That’s a defensible value judgment, but it is a value-judgment and it is contestable. Many ethicists prefer to measure life years (adjusted for quality or not^38^) rather than lives, and doing so generates different predictions and different policy guidance, because a large proportion of those who die with COVID-19 have their lives shortened by a relatively small amount (in contrast, the harms stemming from a recession may hit younger and healthier people more heavily, because wealth is concentrated in older groups in many countries). Both the policy of weighting lives equally and the policy of weighting life years equally are egalitarian policies. Deciding between them is not a matter for epidemiologists. Rather, ethical expertise (among others) is required. General lockdowns, too, embody value judgments: among others, that the burdens should fall equally on all. If the benefits flow disproportionately to older citizens, this judgment is contestable. We do not advocate particular values here. Rather, we emphasize that value judgments are inescapable and that epidemiologists are not best placed to makethem.

It is not merely scientific uncertainties that distinguish climate science (for example) from the science of COVID-19. It is (also) the fact that the consensus in the former has been tested from multiple angles, with experts in fields beyond the sciences having their say and questioning the assumptions used by the climate sciences. There is no such thing as entirely value free science,^39^ but some sciences are more value-laden than others and some values are more contestable than others. Insofar as science serves as an input into public policy, qualifications in science alone are not sufficient for good practice: the value-laden assumptions must be assessed. That has indeed occurred with regard to climate science, but it has not begun with regard to the pandemic. As a consequence, philosophers and ethicists have a much smaller role to play in climate science now and ought instead (very largely) to defer to the experts. But the coronavirus pandemic is not settled science, and we have a role to play in questioning central aspects of government response to the pandemic.

In fact we—and the many others whose expertise is essential to translating epidemiology into public health policy—do not merely have a right to question the science; we have an obligation to do so. It is only when the costs and benefits of lockdowns have been properly assessed that we will possess the knowledge for confident decision-making, and that requires input from many different disciplines. Obviously, economists must play a role. But so must mental health professionals (to assess the costs of isolation, versus those of fear of disease, for example), sociologists (to assess the downstream effects of lockdowns and the broad social changes they bring on child development, for example), educators (to assess the effects of schooling via Zoom), and many others. We must play our different roles. When there is a reliably generated consensus, we ought to defer; right now, we can play our part best in helping to generate that consensus.

Epistemic responsibility imposes different duties on different people in different contexts. Individuals who lack expertise are often epistemically irresponsible when they reject or second guess the conclusions of those who have it. Instead, deference is called for in the face of settled science. But on matters of public policy, expertise is broadly distributed. Many different kinds of people can and should play a role in stress testing such policies. Philosophers, ethicists, lawyers, economists, sociologists, and people in many other disciplines should defer to the expertise of epidemiologists on matters specific to that field but can and should question the policy prescriptions that result. Only through bringing to bear broad ranging expertise can a reliable consensus emerge.

## Footnotes

1
John  Martin  Fischer & Mark  Ravizza, Responsibility and  Control: A Theory of  Moral  Responsibility (2000); Alfred Mele, *Moral Responsibility for Actions: Epistemic and Freedom Conditions*, 13 Philos. Explor. 101–111 (2010); Philip  Robichaud & Jan  Willem  Wieland, Responsibility—The  Epistemic  Condition (2017).

2 Aaron Reeves, Martin McKee & David Stuckler, *Economic Suicides in the Great Recession in Europe and North America*, 205 Br. J. Psychiatry 246–247 (2014).

3 Mahiben Maruthappu et al., *Economic Downturns, Universal Health Coverage, and Cancer Mortality in High-Income and Middle-Income Countries, 1990–2010: A Longitudinal Analysis*, 388 The  Lancet 684–695 (2016).

4 Samantha K. Brooks et al., *The Psychological Impact of Quarantine and How to Reduce It: Rapid Review of the Evidence*, 395 The  Lancet 912–920 (2020).

5 C. A. J. Coady, Testimony: A Philosophical  Study (1992); Jennifer  Lackey & Ernest  Sosa, The  Epistemology of  Testimony (2006).

6 We certainly should avoid idealizing this kind of epistemic outsourcing. Government reports are often prepared by staff who often lack sufficient expertise to distinguish higher from lower quality evidence. They may also lack resources (Matthias Fritsch (*Do Regulators Use Science in Policy Formulation? Evidence from Regulatory Impact Assessments in Australia, New Zealand and the European Union*. Presented at the International Conference on Public Policy, June 26 2019, Montreal, Canada) provides evidence that open access sources were much more likely to be cited than those behind a paywall, because researchers in government lacked library access and often research skills). Notoriously, too, special interests may distort the conclusions reached. This distortion need not arise from corruption, nor even the legal but ethically dubious practice of hiring former politicians to lucrative sinecures soon after retirement. O’Connor & Weatherall (*The Misinformation Age: How False Beliefs Spread* (2019)) use formal modelling to show how selective exposure to carefully curated (but genuine) science can lead policy makers to become more and more convinced of an incorrect claim even as the scientific community converges on the truth.

7 Rebecca Kukla, *“Author TBD”: Radical Collaboration in Contemporary Biomedical Research*, 79 Philos. Sci. 845–858 (2012).

8 Elizabeth Anderson, *Democracy, Public Policy, and Lay Assessments of Scientific Testimony*, 8 Episteme 144–164 (2011); Alvin I. Goldman, *Experts: Which Ones Should You Trust?*, 63 Philos. Phenomenol. Res. 85–110 (2001); Alexander Guerrero, *Living with Ignorance in a World of Experts*, *in*  Perspectives on  Ignorance  From  Moral and  Social  Philosophy 168–197 (2017).

9 Hugo Mercier & Dan Sperber, *Why Do Humans Reason? Arguments for an Argumentative Theory*, 34 Behav. Brain  Sci. 57–74 (2011).

10
Paul  Harris, Trusting  What  You’re  Told (2012); Olivier Mascaro & Dan Sperber, *The Moral, Epistemic, and Mindreading Components of Children’s Vigilance Towards Deception*, 112 Cognition 367–380 (2009).

11 Of course, there may be difficulties for both governments and individuals in accessing reliable information. Oreskes and Conway (Naomi  Oreskes & Erik M. Conway, Merchants of  Doubt: How a  Handful of  Scientists  Obscured the  Truth on  Issues from  Tobacco  Smoke to  Global  Warming (2011)) describe how the false impression that a particular question remains open to doubt can be generated. Equally, an individual may simply be unlucky in choosing a GP and therefore come to trust someone who is an anti-vaxxer. We set these difficult issues aside here, because while misinformation about the coronavirus constitutes a serious problem, we want to focus on the unique problem it poses for experts outside medicine.

12 John Cook et al., *Consensus on Consensus: A Synthesis of Consensus Estimates on Human-Caused Global Warming*, 11 Environ. Res. Lett. 048002 (2016).

13 Daniel F. Gudbjartsson et al., *Spread of SARS-CoV-2 in the Icelandic Population*, 0 N. Engl. J. Med. 1–14 (2020). As a reviewer for this journal reminds us, there have also been some ‘natural’ experiments, such as the Princess Diamond cruise ship and the USS Theodore Roosevelt, which seem to indicate that a higher proportion of the infected remain asymptomatic (see Kenji Mizumoto et al., *Estimating the asymptomatic proportion of coronavirus disease 2019 (COVID-19) cases on board the Diamond Princess cruise ship, Yokohama, Japan, 2020*, 25 Eurosurveillance 2000180 (2020)). Extrapolation from this data is difficult, because (as the reviewer also notes) the virus appears to affect different populations differently.

14 Stefania Boccia, Walter Ricciardi & John P. A. Ioannidis, *What Other Countries Can Learn From Italy During the COVID-19 Pandemic*, JAMA Intern. Med. (2020), https://jamanetwork.com/journals/jamainternalmedicine/fullarticle/2764369 (accessed May 10, 2020).

15 Graphs and Maps from EUROMOMO, https://euromomo.eu/dev-404-page/ (accessed May 10, 2020).

16 Tom Jefferson et al., *Physical Interventions to Interrupt or Reduce the Spread of Respiratory Viruses*, 2011 Cochrane  Database  Syst. Rev. (2011), https://www.ncbi.nlm.nih.gov/pmc/articles/PMC6993921/ (accessed Apr. 3, 2020).

17 Jeffrey Brainard, 2020 & 12:30 Pm, *Surveys of Infectious Disease Experts Aim to Predict COVID-19’s Toll*, Science, 2020, https://www.sciencemag.org/news/2020/04/surveys-infectious-disease-experts-aim-predict-covid-19-s-toll (accessed May 10, 2020).

18 Marta Paterlini, *‘Closing Borders is Ridiculous’: The Epidemiologist Behind Sweden’s Controversial Coronavirus Strategy*, 580 Nature 574–574 (2020).

19 Kathy Leung et al., *First-Wave COVID-19 Transmissibility and Severity in China Outside Hubei After Control Measures, and Second-Wave Scenario Planning: A Modelling Impact Assessment*, 395 The  Lancet 1382–1393 (2020).

20 John P. A. Ioannidis, *Coronavirus Disease 2019: The Harms of Exaggerated Information and Nonevidence-Based Measures*, 50 Eur. J. Clin. Invest. e13222 (2020).

21 Steve Clarke, *Future Technologies, Dystopic Futures and the Precautionary Principle*, 7 Ethics  Inf. Technol. 121–126 (2005); Julian J. Koplin, Christopher Gyngell & Julian Savulescu, *Germline Gene Editing and the Precautionary Principle*, 34 Bioethics 49–59 (2020).

22 Report 12—The Global Impact of COVID-19 and Strategies for Mitigation and Suppression (2020), http://www.imperial.ac.uk/medicine/departments/school-public-health/infectious-disease-epidemiology/mrc-global-infectious-disease-analysis/covid-19/report-12-global-impact-covid-19/ (accessed Apr. 3, 2020).

23 Candice M. Mills & Frank C. Keil, *Knowing the Limits of One’s Understanding: The Development of an Awareness of an Illusion of Explanatory Depth*, 87 J. Exp. Child  Psychol. 1–32 (2004); Leonid Rozenblit & Frank Keil, *The Misunderstood Limits of Folk Science: An Illusion of Explanatory Depth*, 26 Cogn. Sci. 521–562 (2002).

24 Frank C. Keil & Robert A. Wilson, *The Shadows and Shallows of Explanation*, *in*  Minds and  Machines 137–159 (Frank C. Keil & Robert A. Wilson eds, 2000); Nathaniel Rabb, Philip M. Fernbach & Steven A. Sloman, *Individual Representation in a Community of Knowledge*, 23 Trends  Cogn. Sci. 891–902 (2019).

25 Neil Levy, *Radically Socialized Knowledge and Conspiracy Theories*, 4 Episteme 181–192 (2007).

26
Jonathan  Matheson, The  Epistemic  Significance of  Disagreement (2015).

27
Cass R. Sunstein, Infotopia: How  Many  Minds  Produce  Knowledge (2006).

28 Coronavirus Spread Accelerates Again in Germany, Reuters, May 10, 2020, https://uk.reuters.com/article/uk-health-coronavirus-germany-cases-idUKKBN22M01K (accessed May 10, 2020).

29 Edward White & Jung-a Song, *Seoul Restarts Social Distancing After Virus Cluster in Party District*, Financial  Times, October 5, 2020, https://www.ft.com/content/f3ea46c4-e9e5-4163-91a5-c3c5d8f2311c (accessed May 10, 2020).

30 Coronavirus Spread Accelerates Again in Germany, Reuters, May 10, 2020, https://uk.reuters.com/article/uk-health-coronavirus-germany-cases-idUKKBN22M01K (accessed May 10, 2020).

31 Elizabeth Anderson, *Democracy, Public Policy, and Lay Assessments of Scientific Testimony*, 8 Episteme 144–164 (2011); Alvin I. Goldman, *Experts: Which Ones Should You Trust?* 63 Philos. Phenomenol. Res. 85–110 (2001).

32 P.-A Chiappori, S. Levitt & T. Groseclose, *Testing Mixed-Strategy Equilibria When Players Are Heterogeneous: The Case of Penalty Kicks in Soccer*, 92 Am. Econ. Rev. 1138–1151 (2002).

33 Amos Tversky & Daniel Kahneman, *Loss Aversion in Riskless Choice: A Reference-Dependent Model*, 106 Q. J. Econ. 1039–1061 (1991).

34
George  Ainslie, Breakdown of  Will (2001).

35
Daniel  Kahneman, Thinking, Fast and  Slow (2011).

36 Eric Schliesser & Eric Winsberg, *Climate and Coronavirus: The Science is not the Same*, New  Statesman, 2020, https://www.newstatesman.com/politics/economy/2020/03/climate-coronavirus-science-experts-data-sceptics (accessed Apr. 17, 2020).

37 Among others, see John Broome, *Climate Change: Life and Death*, *in*  Climate  Change and  Justice 184–200 (Jeremy Moss ed., 2015); Dale  Jamieson, Reason in a  Dark  Time: Why the  Struggle  Against  Climate  Change  Failed—and  What  It  Means for  Our  Future (2014); Climate  Modelling: Philosophical and  Conceptual  Issues, (Elisabeth A. Lloyd & Eric Winsberg eds., 1st ed. 2018 edn, 2018).

38 See, for discussion, John  McKie et al., The  Allocation of  Health  Care  Resources: An  Ethical  Evaluation of the “QALY” Approach (1998); Peter J. Neumann, Joshua T. Cohen & Milton C. Weinstein, *Updating Cost-Effectiveness—The Curious Resilience of the $50,000-per-QALY Threshold*, 371 N. Engl. J. Med. 796–797 (2014).

39 Heather Douglas, *Inductive Risk and Values in Science*, 67 Philos. Sci. 559–579 (2000).

